# Prevalence and Incidence Rate of Diabetes, Pre-diabetes, Uncontrolled Diabetes, and Their Predictors in the Adult Population in Southeastern Iran: Findings From KERCADR Study

**DOI:** 10.3389/fpubh.2021.611652

**Published:** 2021-11-01

**Authors:** Hamid Najafipour, Maryam Farjami, Mojgan Sanjari, Raheleh Amirzadeh, Mitra Shadkam Farokhi, Ali Mirzazadeh

**Affiliations:** ^1^Cardiovascular Research Center, Institute of Basic and Clinical Physiology Sciences, and Department of Physiology and Pharmacology, Kerman University of Medical Sciences, Kerman, Iran; ^2^Physiology Research Center, Institute of Neuropharmacology, Kerman University of Medical Sciences, Kerman, Iran; ^3^Endocrinology and Metabolism Research Center, Institute of Basic and Clinical Physiology Sciences, and Department of Endocrinology, Kerman University of Medical Sciences, Kerman, Iran; ^4^Social Determinants of Health Research Center, Institute for Futures Studies in Health, Kerman University of Medical Sciences, Kerman, Iran; ^5^Department of Epidemiology and Biostatistics, University of California, San Francisco, San Francisco, CA, United States

**Keywords:** diabetes mellitus, pre-diabetes, undiagnosed diabetes, uncontrolled diabetes, HbA1c, incidence rate, Iran

## Abstract

**Background:** Diabetes mellitus is among the most serious health challenges worldwide. We assessed the prevalence of pre-diabetes (pre-DM) and diabetes (DM), the effectiveness of diabetes management, the 5-year incidence rate, and associated variables in the adult population in southeastern Iran.

**Methods:** In a random cluster household survey (2014–2018), 9,959 adult individuals aged 15–80 years were assessed for coronary artery disease risk factors, including diabetes mellitus in Kerman (KERCADRS, phase 2). Among these people, 2,820 persons had also participated in phase 1 of the study 5 years earlier (2009–2011). Univariable and multivariable survey logistic regression models were used to identify the potential predictors of diabetes and pre-diabetes.

**Results:** The prevalence of pre-DM was 12% (males 13.2% vs. females 11.1%), steadily increasing from 7.1% in the 15–24 years group to 18.4% in the 55–64 years group. The prevalence of DM was 10.2% (male and female, 7.9 and 10.8%, respectively), of which 1.9% were undiagnosed. DM was diagnosed in 10.6% of educated and 15.1% of illiterate people. The prevalence of diagnosed DM was lower in smokers (5.2 vs. 8.7%) and dependent opium users (5.4 vs. 8.8%). The prevalence of uncontrolled DM (HbA1c > 7%) was 48.8%, increasing with age. The frequency of uncontrolled DM among people without and with treatment was 32 and 55.9%, respectively. Illiterate people had worse uncontrolled DM (55.6 vs. 39.6%). The 5-year incidence rate (persons/100 person-years) was 1.5 for pre-DM and 1.2 for DM, respectively. The lowest and the highest incidence rate of DM belonged to the 15–34 years old group (0.5) and dependent opium users (2.4). The incidence rate was found to have a direct relationship with BMI and a reverse relationship with physical activity.

**Conclusion:** Pre-DM and DM affected 22.2% of the population. One-third of patients with diabetes had undiagnosed DM, and in 55.9% of people with diagnosed DM, treatment had been ineffective. Appropriate health interventions are needed to reduce the prevalence and health consequences of diabetes in the region.

## Introduction

Type 2 diabetes mellitus (T2DM) is widely associated with an increased prevalence of cardiovascular disease (CVD) (1, 2). In fact, T2DM patients have a 2- to 4-fold higher risk for CVD morbidity and mortality than healthy non-diabetic patients (3). In addition, accounting for almost 80% of deaths among T2DM patients, CVD is the leading cause of mortality in people suffering from DM (4, 5). The association between T2DM and CVD is not only supported by meta-analyses and observational data (6, 7) but is also based on the pathophysiological background characterizing T2DM on the CV continuum (8, 9). T2DM involves a chronic state of vascular inflammation and endothelial and platelet dysfunction induced by hyperglycemia and insulin resistance, which predisposes the patient to macro-vascular complications even before T2DM is diagnosed (10). It has been reported that coronary plaques with larger necrotic cores and increased inflammation (with more T lymphocytes and macrophages) in addition to a higher rate of plaque ruptures and positive remodeling are generally observed in T2DM patients with non-diabetic controls, suggesting a more active atherosclerotic process (11, 12). The prevalence of diabetes for all age groups worldwide was estimated to be 2.8% in 2000 and 4.4% in 2030 (13). According to the latest data published in the International Federation of Diabetes Atlas, 463 million adults live with diabetes (14). This is an important contributor to disease burden, particularly in developing countries (15).

According to the national CAD risk factors surveillance report, the overall prevalence of diabetes in Iran were estimated to be 8.7% in people aged 15–64 years, about half (4.1%) of whom were newly diagnosed cases (16). Based on a systematic review, the prevalence of T2DM in Iran was estimated as one out of four among adults aged ≥ 40 years (17). However, it is not clear how many are in the pre-diabetic stage, are prone to developing diabetes, and need timely interventions to avoid developing it.

In addition to late diagnosis, diabetes management is a challenging issue in Iran, as only 39.2% of individuals with diagnosed diabetes receive treatment (18). Taking fasting plasma glucose ≥ 130 mg/dl as the criterion for poor management of diabetes, it was found that about 57% of individuals with diagnosed diabetes had a high level of plasma glucose (19).

In a population-based research named the Kerman Coronary Artery Diseases Risk Factors study (KERCADRS) from 2009 to 2011 on 5,900 adults aged 15–75 in Kerman, the prevalence was found to be 18.7% (23.4% men and 13.7% women) for pre-diabetes and 9.0% (7.7% men 10.3% women) for diabetes (20). The present study is the second phase of the KERCADRS performed on a larger sample size of 9,959 to determine the prevalence and predictors of pre-diabetes and diabetes in the adult population aged 15–80 living in an urban setting in southeastern Iran. The results of this study are compared with the findings of the first phase to explore the trend of changes in prevalence and the 5-year incidence rate of pre-diabetes and diabetes. This will provide a better insight into the severity and growth rate of these important CVD risk factors in this region in the past 5 years. We also assessed the effectiveness of diabetes management (using Hb1C as the indicator) in people with diagnosed diabetes. The prevalence of the main CAD-related comorbidities is also reported in normal, pre-diabetic, and diagnosed, and undiagnosed diabetic subpopulations.

## Methods

KERCADRS, the Kerman Coronary Artery Disease Risk Factors Study, is a population-based cohort study with multiple surveys. The study was conducted according to the Declaration of Helsinki, and the study protocols were approved by the Ethics Committee of Kerman University of Medical Sciences (ethics code: IR.KMU.REC.1392.405). Between 2014 and 2018, 9,959 15- to 80-year-old individuals were recruited through a non-proportional-to-size one-stage cluster sampling into the second stage of the study. The methodology of the KERCADR study (phase 1) has been explained in detail elsewhere (21). In brief, 420 zip codes were randomly selected in phase 2, each representing a house (called a seed). The seed household and other households in the neighborhood from the right direction of the seed household were then systematically approached by social mobilizers, and all eligible people (15- to 80-year-olds) in the household were invited to participate in the study. The recruitment continued until 24 persons (12 men and 12 women who provided written informed consent) in each cluster were recruited, reaching a total target sample size of 10,000. For participants under 18, informed consent was acquired from both themselves and their parents, and they usually attended the interview site accompanied by their parents.

### Interview and Measurements

Details on what was measured and how this was achieved are presented elsewhere (21). In brief, trained interviewers assessed the study subjects for different CAD risk factors using a structured questionnaire including questions on demographic information, cigarette smoking (yes/no), opium addiction (no/occasional/dependent), level of physical activity (low/moderate/high), and level of depression and anxiety (BECK questionnaire). The subjects were also asked about their medical and familial history of DM, and whether they were under insulin or non-insulin treatment.

Physical activity was determined by the Global Physical Activity Questionnaire, and the intensity of physical activities was expressed using metabolic equivalents of task (MET). The total MET in time (min) was computed for the status of the activity in work-, transport-, and recreation-related physical activity and was then categorized into three levels of low, moderate, and intense. MET is defined as the rate of energy use in a person in a sitting position (equivalent to 3.5 ml oxygen consumption/kg body weight per minute). Moderate physical activity is the consumption of four times, and intense physical activity is the consumption of eight or more times the energy consumed while sitting (22). Hypertension was defined as systolic blood pressure ≥ 140 mmHg or diastolic blood pressure ≥ 90 mmHg, or taking any antihypertensive drug. Body mass index (BMI) between 25 and 29.9 kg/m^2^ and BMI ≥ 30 kg/m^2^ were interpreted as overweight and obese, respectively.

Diabetes was diagnosed according to the ADA recommendation (23). Every individual who had previously been diagnosed with diabetes (by a physician) or was taking insulin or oral anti-diabetic medications or had FPG ≥ 126 mg/dl at the time of recruitment (provided that the HbA1c was more than 6.5%) was considered diabetic. Those with FPG between 100 and 125 mg/dl were considered pre-diabetic (pre-DM). Participants with FPG ≥ 126 mg/dl for the first time were called back, and HbA1c was checked, and if HbA1c was more than 6.5%, diabetes was confirmed. None of the patients had GLP-1 agonists as a treatment option.

Those with FPG ≥ 126 and HbA1c lower than 6.5% were not included as diabetics (these were 46 subjects vs. 1,020 individuals with diabetes, see study limitations). Subjects who had no previous history of diabetes or anti-diabetic medication but were found to have FPG ≥ 126 mg/dl and HbA1c higher than 6.5% on recruitment were considered undiagnosed (new) diabetic cases (20). Every diabetic case was tested for HbA1c to determine the glycemic control status of diagnosed patients with diabetes.

Based on ADA recommendation, “Older adults who are otherwise healthy with few coexisting chronic illnesses and intact cognitive function and functional status should have lower glycemic goals [such as HbA1C 7.5% (58 mmol/mol)], while those with multiple coexisting chronic illnesses, cognitive impairment, or functional dependence should have less stringent glycemic goals [such as HbA1c 8.0–8.5% (64–69 mmol/mol)]” (23).

Uncontrolled diabetes was specified as HbA1c > 7%, and in people with a progression of microvascular and chronic complications and those aged more than 70, HbA1c > 8% was considered the cut-off for poor glycemic control (uncontrolled DM).

### Laboratory Measurements

All participants were asked to fast for 12 h before coming to the clinic. Venous blood samples were obtained from the antecubital vein between 7:00 and 9:00 a.m., and fasting plasma glucose (FPG) was measured in serum (Kimia Kit, Code 890410, Iran). Subjects with diagnosed diabetes and new cases with FPG higher than 100 mg/dl were called back for another FPG and HbA1c test (NycoCard Kit, Code 1042184, Austria). Serum lipids for all participants were measured using KIMIA Kit; total cholesterol was assessed using KIMIA Kit, Code 890303, Iran, while triglycerides were assessed using KIMIA Kit, Code 890201, Iran.

### Incidence Rate of Diabetes/Pre-Diabetes

**Figure 1:**
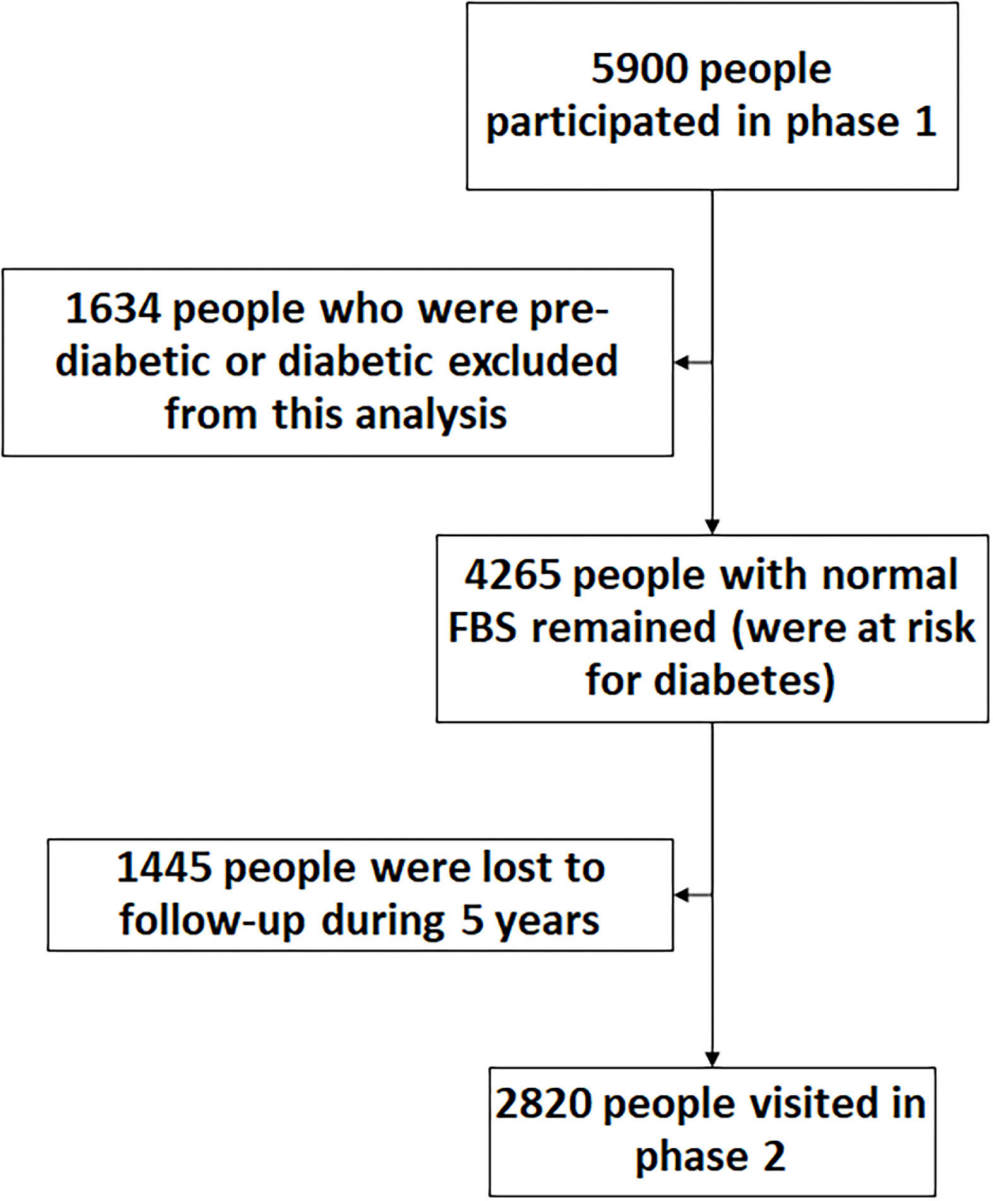
The flow chart of people participated in both phases of the study.

We used the same method to calculate the incidence rate for pre-diabetes and diabetes. Therefore, we only present the method for measuring the latter here. To calculate the incidence rate of diabetes, we used the data from those who had participated in both phases, had normal FBS with no anti-diabetes treatment in phase 1, and, therefore, were at risk of becoming diabetic during the follow-up (Figure 1). Therefore, 27.7% of the 5,900 participants (1,634 cases) in phase 1 who were already pre-diabetic/diabetic were excluded from the incidence calculation. Out of the remaining participants (4,265 cases), 1,445 persons (24.5% of total participants) were lost to follow-up (did not take part in Phase 2 or had died). The number of new diabetic cases (among 4,265 cases) identified during the follow-up period was considered the numerator. The time difference (in years) between the visit in phase 1 and the visit in phase 2 was calculated as person-year at risk for those who had normal FPG in the phase 1 visit. Therefore, the denominator is the sum of the time each person was followed (person-year), totaled for all 4,265 persons at risk of becoming diabetic. For those lost to follow-up, on average, 2.5 years (half of overall follow time) was taken as years at risk. Then incidence rate (expressed as person per 100 person-years) was calculated by the formula (24):


$$\begin{array}{l} \text{Incidence rate}\\ =\frac{\text{No}.\text{ of new cases of DM during 5 years follow up}}{\text{Total person}-\text{years for all persons at risk of DM}}\times 100 \end{array}$$

### Statistical Analysis

**Figure 2:**
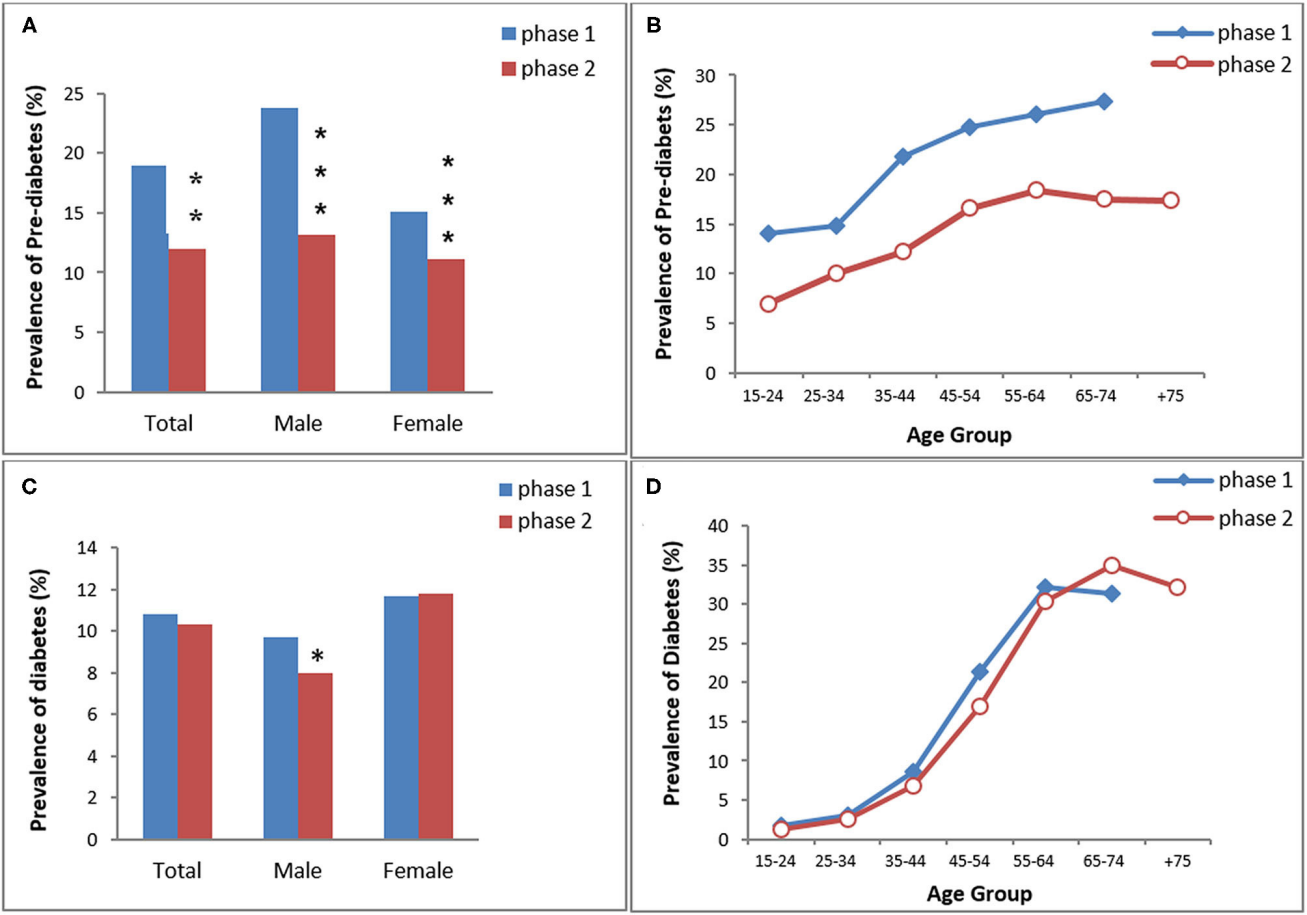
The prevalence of pre-diabetes **(A,B)** and diabetes **(C,D)** in KERCADRS by sex and age groups. Total participants = 9,959 in phase 2 and 5,900 in phase 1. The data of phase 1 were used here for comparison and are extracted from our paper published previously (20). *P < 0.05, **P < 0.01, ***P < 0.001 compared to phase 1.

**Figure 3:**
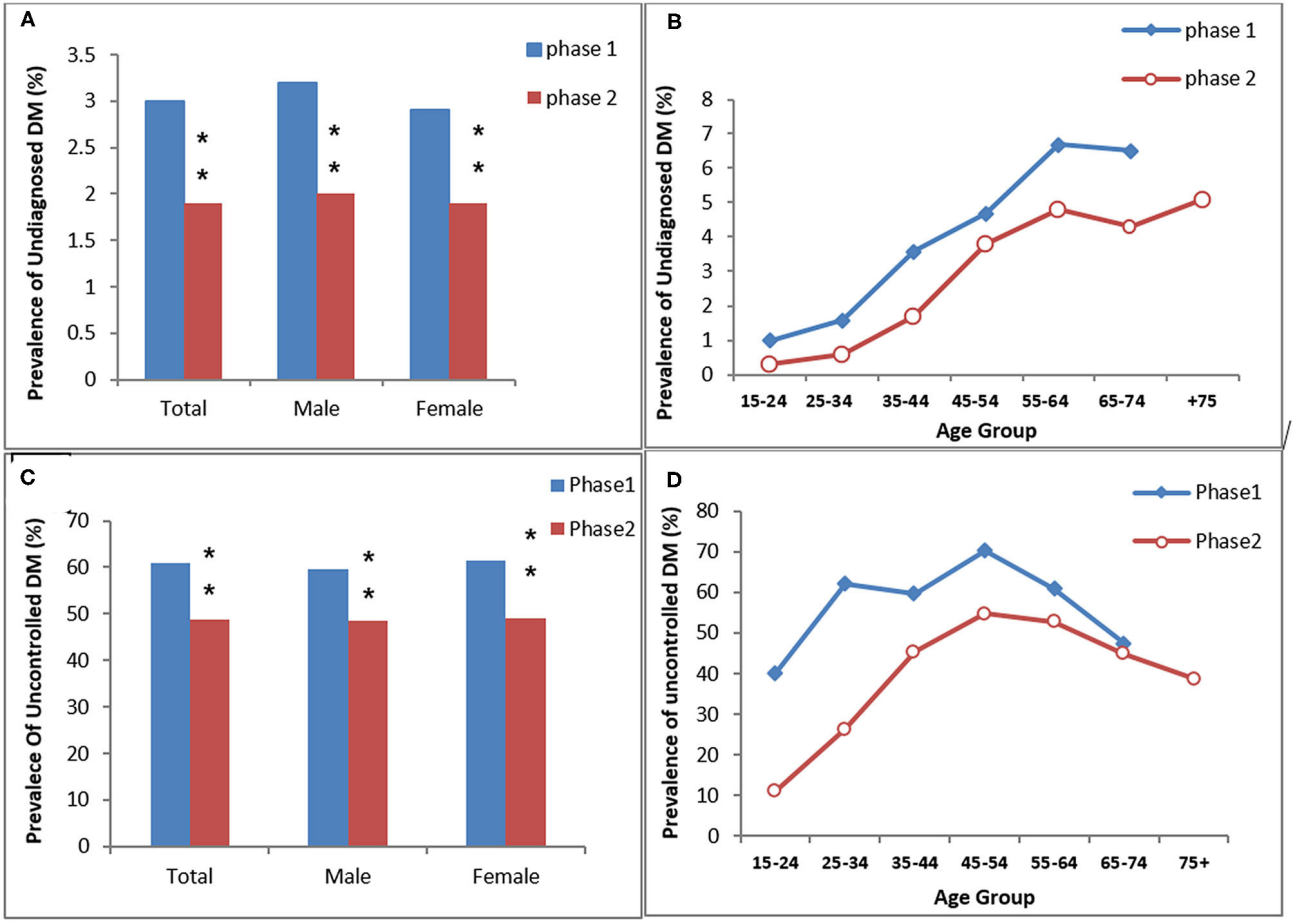
The prevalence of undiagnosed DM **(A,B)** and uncontrolled DM in the diagnosed diabetic participants **(C,D)** in the study (KERCADRS) by sex and age groups. Total participants were 9,957 in phase 2 and 5,900 in phase 1. The data of phase 1 were used here for comparison and are extracted from our paper published previously (20). **P < 0.01 compared to phase 1.

All statistical analyses were conducted under survey data analysis using Stata v. 15 (Stata Corp. 2015 College Station, Texas USA). Data are presented as absolute and relative frequencies and 95% confidence intervals (95% CI). To account for the clustering effect, we used the survey data package analysis, in which we set clusters to be the primary sampling units. Then, because of the non-proportionate-to-size sampling method, the total estimates were standardized based on the real age distribution of the target population (national census of Kerman population size in 2016). We reported weighted prevalence (25) for pre-DM and DM. We ran the bivariate analysis to assess the association between all covariates and the study outcome [pre-DM and DM (both diagnosed and undiagnosed) binary outcomes] one at a time. Then, we included all covariates with *P*-values < 0.05.n (based on likelihood test) in the multivariable logistic regression. Outputs from univariable and multivariable survey logistic regressions were reported as crude and AOR. Data from the second phase of the study were used in the logistic regression. *Z*-test and the Chi-square test were used to compare the prevalence between phases 1 and 2 (Figures 2, 3).

## Results

### Demographic Characteristics

The 9,959 people recruited in this study had a median age of 47 years with an interquartile range (IQR) of 24 years, and 59.4% were female. There was no significant difference between females (median 46, IQR 23 years) and males (median 47, IQR 26 years) in this regard. According to the self-report, 5.2% had never been to school, and one-third (33.5%) had not completed secondary education (had not finished high school).

### Pre-DM and DM Prevalence

Overall, the age- and sex-standardized prevalence of pre-diabetes (pre-DM) was 12.0% (men 13.2% vs. women 11.1%) (Table 1). The pre-DM prevalence showed an increasing trend from 7.1% in young adults (age group 15–24 years) to 18.4% in middle-aged people (age group 55–64 years). Almost 19% of illiterate people were pre-DM, while the prevalence was lower in people with above-high-school education (11.5%). 8.9% of people with normal BMI, 13.1% of those with overweight, and 15.8% of obese individuals had pre-DM (*P* < 0.01). Smoking, opium use, the level of physical activity, and having a familial history of DM had no significant effect on the prevalence of pre-DM.

**Table 1:** The standardized prevalence % (95% confidence interval CI) of pre-diabetes, undiagnosed and diagnosed diabetes, and adjusted odds ratio for different predictors of diabetes mellitus; community-based cohort study (KERCADRS-2nd Phase-*N* = 9,959), Kerman, Iran 2014–2018.

**Subgroups**	**Pre-DM (*n* = 1,415)**	**Undiagnosed DM (*n* = 286)**	**Diagnosed DM (*n* = 1,357)**	**Adjusted OR for DM (95% CI)**	***P*-value**
**Overall**	12.0 (11.3–12.7)	1.9 (1.7–2.2)	8.3 (7.9–8.8)	–	–
**Sex**
Male	13.2 (12.0–14.3)	1.9 (1.5–2.3)	6.0 (5.4–6.6)	1	
Female	11.1 (10.3–12.0)	1.9 (1.6–2.2)	9.9 (9.2–10.5)	1.13 (0.98–1.32)	0.084
**Age group (years)**
15–24	7.1 (5.8–8.3)	0.3 (0.06–0.6)	1.0 (0.5–1.4)	1	
25–34	10.0 (9.0–11.1)	0.6 (0.4–0.9)	1.9 (1.5–2.4)	1.57 (0.82–2.99)	0.169
35–44	12.2 (11.1–13.2)	1.7 (1.3–2.1)	5.1 (4.4–5.7)	3.62 (1.98–6.60)	<0.001
45–54	16.6 (15.4–17.7)	3.8 (3.2–4.4)	13.0 (12.0–14.0)	10.11 (5.61–18.19)	<0.001
55–64	18.4 (17.2–19.6)	4.8 (4.1–5.4)	25.5 (24.2–26.9)	23.49 (13.09–42.16)	<0.001
65–74	17.5 (15.8–19.2)	4.3 (3.4–5.3)	30.7 (28.6–32.7)	30.05 (16.5–54.4)	<0.001
75	17.4 (14.5–20.2)	5.1 (3.5–6.8)	27.0 (23.6–30.3)	30.21 (16.06–56.8)	<0.001
**Education**
Illiterate	18.9 (10.7–27.0)	6.6 (0.5–12.7)	15.1 (9.8–20.4)	1	
Primary to high school	14.7 (13.8–15.5)	2.8 (2.4–3.2)	13.0 (12.2–13.8)	0.92 (0.76–1.1)	0.36
Above high school	11.5 (9.9–13.1)	2.1 (1.4–2.8)	10.6 (9.0–12.2)	0.70 (0.55–0.89)	<0.004
**Current cigarette smoker**
No	12.0 (11.3–12.7)	1.9 (1.7–2.2)	8.7 (8.2–9.2)	1	
Yes	11.9 (9.0–14.8)	1.7 (1.0–2.4)	5.2 (3.8–6.6)	0.70 (0.55–0.89)	<0.005
**Opium use**
No	11.8 (11.0–12.5)	1.9 (1.6–2.2)	8.8 (8.3–9.3)	1	
Occasional	14.6 (9.9–19.3)	1.7 (1.1–2.4)	6.6 (5.4–7.8)	0.99 (0.82–1.18)	0.91
Dependent	12.8 (9.4–16.3)	1.3 (0.5–2.1)	5.4 (3.1–7.6)	0.77 (0.53–1.12)	0.18
**Depression**
No	12.3 (11.5–13.0)	2.1 (1.8–2.3)	7.9 (7.4–8.3)	1	
Yes	10.4 (8.7–12.1)	1.1 (0.7–1.5)	10.7 (9.4–12.0)	1.04 (0.88–1.2)	0.62
**Anxiety**
No	12.5 (11.6–13.4)	2.0 (1.7–2.3)	7.3 (6.8–7.9)	1	
Yes	11.3 (10.3 12.3)	1.7 (1.3–2.1)	9.7 (8.9–10.4)	1.11 (0.97–1.2)	0.1
**Body mass index**
Normal	8.9 (8.0–9.9)	1.0 (0.7–1.3)	6.1 (5.4–6.8)	1	
Overweight	13.1 (11.8–14.5)	2.1 (1.7–2.6)	8.0 (7.4–8.7)	1.37 (1.17–1.60)	<0.001
Obese	15.8 (13.7–17.9)	2.6 (2.0–3.2)	11.5 (10.2–12.8)	1.77 (1.5–2.09)	<0.001
**Family history of DM**
No	11.8 (10.9–12.7)	1.6 (1.3–1.9)	5.7 (5.2–6.2)	1	
Yes	11.9 (10.7–13.0)	2.3 (1.9–2.8)	12.8 (11.9–13.7)	2.5 (2.2–2.8)	<0.001
**Physical activity**
Low	11.5 (10.4–12.7)	2.0 (1.6–2.4)	8.8 (8.2–9.5)	1	
Moderate	12.0 (11.3–12.7)	1.9 (1.5–2.3)	8.6 (7.8–9.3)	1.4 (1.14–1.71)	0.001
High	11.7 (10.0–13.4)	1.6 (1.0–2.2)	5.9 (4.8–6.9)	1.3 (1.10–1.65)	0.004

*CI, confidence interval; DM, diabetes mellitus; OR, odds ratio*.

The age- and sex-standardized prevalence of pre-DM was 18.9% (men 23.8% vs. women 15.1%; *P* < 0.001) in phase 1 and 12% (men 13.2% vs. women 11.1%; *P* < 0.01) in phase 2. Overall, pre-DM in both genders was significantly lower in phase 2 than in phase 1 (*P* < 0.01 between two phases). The prevalence of pre-DM increased with age in both phases and it was lower in phase 2 in all age groups (Figure 2).

The prevalence of DM was 10.8% in phase 1 (men 9.7% vs. women 11.7%) and 10.3% in phase 2 (men 8% vs. women 11.8%) (Figure 2). Only males had a significantly lower prevalence of DM in phase 2 compared to phase 1. There was no difference in age group trends of DM between the two phases.

### Predictors of Diabetes

In the multivariable model, after controlling for confounders, it was shown that odds of diabetes (diagnosed and undiagnosed combined) had an increasing trend by age group (AOR up to 30.2) and had a positive association with familial history of DM (AOR 2.5), overweight and obesity (AOR 1.37 and 1.77), and low physical activity (AOR 1.3) (Table 1). A significant negative association was found between smoking and odds of diabetes (AOR 0.7). Depression, anxiety, and opium use had no statistically significant association with diabetes.

### Undiagnosed and Diagnosed Diabetes

In total, the standardized prevalence of diabetes was 10.2% (men 7.9% and women 10.8%), of which 1.9% had undiagnosed diabetes (equal for men and women), and 8.3% had had their disease already diagnosed (men 6.0% vs. women 9.9%) (Table 1).

Undiagnosed DM was more prevalent in illiterates (6.6%) compared to higher-educated people, in older individuals (5.1% among people over 75) compared to youngers, in obese people (2.6%) compared to those with normal weight, and in those with positive family history of DM (2.3%). People with anxiety symptoms (1.7%), high physical activity (1.6%), and depression (1.1%) had a lower prevalence of undiagnosed DM compared to those with low physical activity and normal mental status. There was an equal prevalence of undiagnosed DM in males and females (Table 1).

The diagnosed-DM showed an increasing trend in the prevalence by age from 1% in young adults (age group 15–24) to 30.7% in older adults (aged 65–74 years). DM was diagnosed in 10.6% of educated (above high school) people compared to 15.1% illiterate participants. Compared to non-cigarette-smokers, the prevalence of diagnosed DM was lower among smokers (5.2 vs. 8.7%). The prevalence of diagnosed DM was 8.8% in non-opium-users, 6.6% in occasional opium users, and 5.4% among dependent users. Eight percent of overweight and 11.5% of obese people had diagnosed DM. In people with high physical activity, 5.9% had diagnosed DM, compared with 8.8% in people with low physical activity. Compared to subjects with negative family history of DM, people with positive history had a higher prevalence of diagnosed DM (12.8 vs. 5.7%) (Table 1).

### Diabetes Mismanagement

Regarding the HbA1c > 7 (old definition) in people with diagnosed DM, the prevalence of uncontrolled DM was 50.2% (men 49.8% vs. women 50.4%) (Table 2). Uncontrolled DM showed an increasing trend in prevalence by age from 11.1% in young adults to 52.7% in older people (15–24 years old and 65–74 years old, respectively). The frequency of uncontrolled DM among people without treatment was 32%, but it was 55.9% in those under treatment (both insulin and oral). Less educated people had more uncontrolled DM than highly educated individuals (58.3 vs. 40.8%). Uncontrolled DM among non-smokers and no-opium users (50.3 and 50.3%) was higher than in smokers and dependent opium users (48.2 and 46.6%). Regarding obesity, uncontrolled DM ranged from 53.1% (normal BMI) to 49.3% (obese subgroup). More than fifty-two percent of patients with diabetes who had a positive history of familial DM were uncontrolled.

**Table 2:** The prevalence % (CI) of un-controlled diabetes among diagnosed diabetes patients (*n* = 1,359), community-based cohort study (KERCADRS 2nd Phase-*N* = 9,959), Kerman, Iran 2014–2018.

**Subgroup**	**Uncontrolled DM (HbA1c > 7)**	**Uncontrolled DM (HbA1c > 8)**
	**(*n* = 683)**	**(*n* = 664)**
**Overall**	50.2 (47.5–52.9)	48.8 (46.1–51.5)
**Sex**
Male	49.8 (45.0–54.7)	48.4 (43.6–53.3)
Female	50.4 (47.1–53.6)	49.0 (45.7–52.2)
**Age group (year)**
15–24	11.1 (0.2–48.2)	11.1 (0.2–48.2)
25–34	26.4 (12.8–44.3)	26.4 (12.8–44.3)
35–44	45.3 (35.7–55.2)	45.3 (35.7–55.2)
45–54	54.7 (48.6–60.6)	54.7 (48.6–60.6)
55–64	52.7 (48.3–57.0)	52.7 (48.3–57.0)
65–74	47.6 (41.9–53.4)	44.7 (39.0–50.5)
75	49.4 (38.9–60.0)	38.7 (28.7–49.3)
**Medical treatment**
None	32.0 (25.8–38.8)	30.1 (24.0–36.8)
Oral	52.5 (49.2–55.7)	50.9 (47.6–54.1)
Insulin	60.3 (51.0–69.1)	60.3 (51.0–69.1)
Insulin and oral	55.9 (45.2–66.2)	55.9 (45.2–66.2)
**Education**
Illiterate	58.3 (52.0–64.4)	55.6 (49.3–61.8)
Primary to high school	49.6 (46.4–52.9)	48.6 (45.3–51.8)
Above high school	40.8 (33.2–48.7)	39.6 (32.0–47.5)
**Current cigarette smoker**
No	50.3 (47.6–53.1)	48.9 (46.1–51.7)
Yes	48.2 (37.4–59.2)	47.1 (36.3–58.1)
**Opium use**
No	50.3 (47.3–53.2)	48.9 (46.0–51.9)
Occasional	51.4 (44.3–58.5)	49.5 (42.4–56.6)
Dependent	46.6 (28.3–65.6)	46.6 (28.3–65.6)
**Depression**
No	50.2 (47.2–53.3)	48.8 (45.8–51.9)
Yes	50.0 (43.9–56.0)	48.5 (42.5–54.6)
**Anxiety**
No	51.0 (47.3–45.7)	49.5 (45.8–53.2)
Yes	49.2 (45.3–53.2)	48.0 (44.0–51.9)
**Body mass index**
Normal	53.1 (47.3–58.8)	51.8 (46.0–57.5)
Overweight	49.4 (45.1–53.7)	47.8 (43.5–52.0)
Obese	49.3 (44.8–53.7)	48.1 (43.6–52.5)
**Family history of DM**
No	47.8 (43.7–51.9)	47.0 (42.9–51.1)
Yes	52.3 (48.6–55.9)	50.9 (47.2–54.6)
**Physical activity**
High	50.3 (41.5–59.2)	48.8 (40.0–57.7)
Moderate	49.3 (44.9–53.7)	48.3 (43.9–52.7)
Low	50.9 (47.1–54.6)	49.2 (45.4–52.9)

*CI, confidence interval; DM, diabetes mellitus*.

Given age and coexisting complications (details are mentioned above in the method), overall, uncontrolled DM (HbA1c > 8%, patient centered) was observed in 48.8% of diagnosed DM cases (men 48.4% vs. women 49%) (Table 2). The prevalence of uncontrolled DM ranged from 0.3 to 43% in different subpopulations. The greatest difference was observed among age groups (43.5%), in those receiving insulin therapy (30%), and in illiterates (16%). Familial history of DM (3.9%), opium use (3.4%), and the level of physical activity (0.4%) have minor association with the status of diabetes control (Table 2). The frequencies of drug-naivety, use of oral agents, insulin monotherapy, and insulin combination therapy were 32.0% (*n* = 219), 60% (*n* = 435) 7.7% (*n* = 56), and 2.1% (*n* = 15) in phase 1 and 15.6% (*n* = 212), 68.7% (*n* = 933), 8.9% (*n* = 121), and 6.8% (*n* = 15) in phase 2, respectively.

The age- and sex-standardized prevalence of undiagnosed DM was 3% (men 3.2% vs. women 2.9%) in phase 1 and 1.9% (men 2% vs. women 1.9%) in phase 2. Both overall and age-dependent prevalence of undiagnosed DM were significantly lower in phase 2 compared to phase 1 of the study (*P* < 0.01 between the two phases, Figures 2A,B). The prevalence of uncontrolled-DM in the diagnosed DM patients was 48.8% (men 48.4% vs. women 49%) in phase 2 and 60.8% (men 59.7% vs. women 61.5%) in phase 1. Uncontrolled DM overall and in both genders was also significantly lower in phase 2 than in phase 1 (*P* < 0.01 between two phases, Figures 3C,D).

### Diabetes-Related Comorbidities

The maximum prevalence of comorbidities among patients with diagnosed DM included overweight/obesity (77.3%), hypertriglyceridemia (70.3%), hypertension (61.0%), and hypercholesterolemia (55.4%) (Table 3). Among people with undiagnosed DM, the most frequent comorbidities were again overweight/obesity (83.5%), hypertriglyceridemia (61.8%), and hypertension (44.7%). Depression had the lowest association with diagnosed DM (20.4%) and undiagnosed DM (10.1%). Among individuals with pre-DM, again, overweight/obesity (75.9%), hypertriglyceridemia (46%), and hypertension (35.6%) were the most frequent comorbidities. The least frequent comorbidity among individuals with pre-DM was depression (13.7%).

**Table 3:** The prevalence % (CI) of different comorbidities in normal, pre-diabetes, and undiagnosed and diagnosed diabetes, community-based cohort study (KERCADR−2nd Phase-*N* = 9,959), Kerman, Iran, 2014–2018.

**Comorbidities (PHASE2)**	**Normal**	**Pre-DM**	**Undiagnosed DM**	**Diagnosed DM**
Hypertension	22.1 (21.2–23.1)	35.6 (33.1–38.1)	44.7 (38.9–50.5)	61.0 (58.4–63.6)
Hypercholesterolemia	14.1 (13.3–15.0)	26.6 (24.3–28.9)	40.5 (34.8–46.2)	55.4 (52.8–58.1)
Hypertriglyceridemia	30.3 (29.2–31.3)	46.0 (43.4–48.6)	61.8 (56.2–67.5)	70.3 (67.9–72.7)
Depression	15.9 (15.0–16.7)	13.7 (11.9–15.5)	10.1 (6.6–13.6)	20.4 (18.3–22.6)
Anxiety	40.6 (39.4–41.7)	39.2 (36.6–41.7)	35.6 (30.1–41.2)	46.1 (43.4–48.7)
Overweight and obesity	59.4 (58.2–60.5)	75.9 (73.6–78.1)	83.5 (79.2–87.8)	77.3 (75.1–79.5)

*CI, confidence interval; DM, diabetes mellitus; pre-DM, pre-diabetes*.

### Incidence Rate of Pre-diabetes and Diabetes

The incidence rate of pre-DM and DM during the 5 years between the two phases of the study is presented in Table 4. Overall, the incidence rate per 100-person years was 1.5 for pre-DM and 1.2 for DM, with a higher incidence rate for men in pre-DM and for women in DM. The lowest incidence rate of pre-DM was among those who had high physical activity (1.2 persons/100 person-years) and in middle-aged (35–44 years old) adult participants (0.7 persons/100 person-years) while the highest incidence rate was observed among those in the age group of 65–74 years old (2.5 persons/100 person-years) and occasional opium users (2.2 persons/100 person-years). Similarly, the lowest incidence rate of DM was seen in young (15–34 years old) adults and cigarette smokers (0.5 and 0.8 persons/100 person-years, respectively) while the highest incidence rate was observed among dependent opium users (2.4 persons/100 person-years and those in the age group of 65–74 years (2.1 persons/100 person-years). There was a reverse relationship between the incidence rate of DM and the level of physical activity and a direct relationship between the incidence rate of DM and BMI (Table 4).

**Table 4:** Age-Sex-specific incidence rate (IR) (person per 100 person-years) for different associated factors of pre-diabetes and undiagnosed and diagnosed diabetes, community-based cohort study (KERCADRS) 1st and 2nd Phases (*n* = 2,820 match cases), Kerman, Iran, phase 1, 2009–2011 and phase 2, 2014–2018.

**Subgroups**	**Pre-DM**			**DM (Diagnosed and Undiagnosed)**		
	**Number of pre-DM**	**Person-years**	**IR of pre-DM (95% CI)**	**Number of DM**	**Person-years**	**IR of DM (95% CI)**
**Overall**	144	9331.66	1.5 (1.3–1.8)	160	12544.66	1.2 (1.0–1.4)
**Sex**
Male	77	4107.39	1.8 (1.4–2.3)	58	5836.71	0.9 (0.7–1.2)
Female	67	5224.27	1.2 (0.9–1.6)	102	6707.94	1.5 (1.2–1.8)
**Age group (year)**
15–24	5	423.79	1.1 (0.3–2.7)	–	–	–
25–34	19	1737.43	1.0 (0.6–1.7)	9	1737.43	0.5 (0.2–0.9)
35–44	15	2003.51	0.7 (0.4–1.2)	14	2482.52	0.5 (0.3–0.9)
45–54	36	2041.10	1.7 (1.2–2.4)	49	2794.90	1.7 (1.2–2.3)
55–64	32	1784.49	1.7 (1.2–2.5)	42	2652.01	1.5 (1.1–2.1)
65–74	23	905.17	2.5 (1.6–3.7)	33	1512.24	2.1 (1.5–3.0)
75	14	414.72	1.4 (0.5–3.1)	13	639.86	2.0 (1.0–3.4)
**Current cigarette smoker**
No	128	8442.14	1.5 (1.2–1.8)	149	11302.01	1.3 (1.1–1.5)
Yes	16	889.52	1.7 (1.0–2.9)	11	1242.65	0.8 (0.4–1.5)
**Opium use**
No	114	7970.78	1.4 (1.1–1.7)	135	10514.68	1.2 (1.0–1.5)
Occasional	25	1097.32	2.2 (1.4–3.3)	23	1684.75	1.3 (0.8–2.0)
Dependent	5	263.57	1.8 (0.6–4.3)	2	81.67	2.4 (0.2–8.5)
**Depression**
No	91	6011.18	1.5 (1.2–1.8)	94	8132.77	1.1 (0.9–1.4)
Yes	53	3320.48	1.5 (1.1–2.0)	66	4406.83	1.4 (1.1–1.9)
**Anxiety**
No	34	2265.93	1.5 (1.0–2.0)	41	3136.04	1.3 (0.9–1.7)
Yes	110	7065.73	1.5 (1.2–1.8)	119	9408.62	1.2 (1.0–1.5)
**Body mass index**
Normal	54	4466.82	1.2 (0.9–1.5)	29	5546.90	0.5 (0.3–0.7)
Overweight	57	3162.85	1.8 (1.3–2.3)	76	4625.92	1.6 (1.2–2.0)
Obese	31	1643.50	1.8 (1.2–2.6)	55	2300.91	2.3 (1.8–3.1)
**Physical activity**
Low	68	3913.81	1.7 (1.3–2.1)	83	5300.95	1.5 (1.2–1.9)
Moderate	65	4553.11	1.4 (1.1–1.8)	69	6076.65	1.1 (0.8–1.4)
High	11	864.74	1.2 (0.6–2.2)	8	1167.06	0.6 (0.2–1.3)

*CI, confidence interval; IR, incidence rate; DM, diabetes mellitus; pre-DM, pre-diabetes*.

## Discussion

Our analysis revealed that one out of four individuals living in the urban area in southeast Iran either had impaired glucose levels (pre-diabetes) or was diabetic. Close to 2% of studied individuals had undiagnosed diabetes, and in about 50% of diagnosed patients with diabetes, the treatment was not effective.

Both the number of cases and the prevalence of diabetes have been steadily increasing over the past few decades, particularly in low- and middle-income countries. We observed an almost equal prevalence of DM in our study population (10.2%) with that reported by WHO in 2016 on the prevalence of DM in Iran (10.3%). However, we found that 13.2% of men and 11.1% of women are pre-diabetic. This should be taken as an opportunity by health authorities to reduce the burden of the disease by preventing pre-diabetic cases from developing the full-blown disease. It has been shown that by losing weight and increasing physical activity, individuals can prevent or delay pre-diabetes from progressing to diabetes (26–29).

Fortunately, the prevalence of pre-diabetes decreased significantly during the 5 years between the two phases of the study in all age groups (Figure 2A) although overall, this reduction was more prominent in males. Also, the prevalence of diabetes has stopped rising. Previously, in urban areas, patients' compliance with medication and regular visits to physicians depended on the patients themselves. About 7 years ago, the family physician program was piloted in a few cities and was then expanded to many urban areas in Iran to cover this gap in primary healthcare in urban settings (30, 31).

We think this study provides evidence of the positive effects of such interventions and the effect of the health reform plan, which was carried out mostly in urban areas of the country during the 5 years between the two phases of the study. The significant reduction in the frequency of drug-naivety and increase in the use of oral agents or insulin therapy in phase 2 compared to phase 1 (see results section under “Diabetes Mismanagement”) is a validation of this hypothesis. Also, the prevalence of undiagnosed DM was significantly reduced in phase 2 compared to phase 1 (Figure 3), verifying the increase in patients' willingness to refer to physicians in the health system.

Unlike pre-diabetes, the prevalence of diabetes showed a slight reduction, only in males, during the 5 years between the two phases of the study (Figure 2B). We hypothesize that the wave of current reduction in the prevalence of pre-diabetes will affect diabetes soon. This should be monitored over time by the next phase of the KERCADRs study or other population-based surveys.

In both phases of the study, the prevalence of diabetes was significantly higher in women than in men, which agrees with the findings of a recent study, which found that females were more affected by diabetes (32). The frequencies of cardiometabolic risk factors were also significantly different in men and women, with diabetes and obesity the more predominant traits among women (33). The higher prevalence among the women can be partly explained by a higher prevalence of obesity (34–36) and lower physical activity reported in several studies (37–39) in them. These findings were different from a recent study in China, where there was no significant gender difference in the prevalence of DM (men, 14.1% and women, 14.5%) (29). There is a slight sex difference in the global numbers of people with diabetes, with an estimated 17 million more diabetic men than women in 2017. The prevalence increases sharply with age in both sexes (40).

It has been reported in several studies that the prevalence of diabetes (diagnosed and undiagnosed), IGT, and IFG increases by age (40, 41). This is a physiologic phenomenon in which the prevalence of metabolic disorders increases with age, especially in women after menopause, when the level of sex hormones decreases sharply. The age dependency of diabetes was found in the present study up to the age of 64. The reduction in the prevalence of diabetes among individuals aged 65 years or more found in the present study could be due to higher mortality in patients with diabetes in comparison to the rest of the population; diabetes has been reported as a trigger for other cardiovascular diseases such as hypertension, stroke, and acute myocardial infarction (42).

In our study, the lowest incidence rate of pre-DM was observed among the middle-aged (35–44 years old) adult participants, while the highest incidence rate was seen among those in the 65–74 years age group. Fiorentino et al. showed that young IGT and adult IGT subjects exhibited a progressively greater degree of hepatic insulin resistance and reduced insulin clearance compared with older IGT subjects (43). The incidence rate was lower in smokers, which seems unexpected (44). However, it has been shown that smoking causes lower BMI and evidence from a systematic review and meta-analysis shows that the risk of type 2 diabetes is raised in new quitters compared with those who have never smoked (45). Also, smoking cessation is associated with deterioration in glycaemia control in smokers with type 2 diabetes (46).

The observed trend of diabetes during the 5-year interval between the study's two phases indicated that both overall and age-dependent pre-diabetes decreased significantly. This, along with the overall stability in the prevalence of diabetes, is promising. Iran has a well-developed primary healthcare system in rural areas, with the “Behvarz” health workers responsible for population-based prevention and control services. The effectiveness of Behvarz health workers in rural areas on better diabetes management (both diagnosis and treatment) has been mentioned (18). Likewise, in recent years, the government has made significant improvements in the primary health care system of urban areas. Unfortunately, based on the results of KERCADRS phase 2, physical activity has decreased, especially in young individuals, during the 5-year interval between the two phases of the study (47), and overweight and obesity have increased in the area. These may decelerate the positive effect of improvements in other health factors.

The other important finding of the study was that the highest incidence rate of pre-DM and DM was related to occasional and dependent opium users. There is a belief among most opium users that this substance will reduce blood glucose (48) and that it is beneficial to patients with diabetes. This study did not verify this belief. On the other hand, the lowest incidence rate of pre-DM and DM was in those with high physical activity, while low physical activity is quite prevalent among opium users (47).

### Strengths and Weaknesses

In addition to the broad age range of the participants and the beneficial and definitive epidemiological information related to CAD risk factors in the population, which the study presented to health authorities, we should acknowledge the limitations of our survey. Firstly, KERCADRS was not an interventional study. Secondly, in the present study, those with FPG ≥ 126 mg/dl were examined for the first time, and HbA1c <6.5% in recruitment were not included as people with diabetes. This happened because we had used HbA1c as the second test for confirming the diagnosis of diabetes. Assessing HbA1c as the second test confirmed the diagnosis and also showed us how diabetes is controlled. As there were 46 patients with the mentioned conditions, assuming that half of these were diabetic, the prevalence of reported diabetes in the present study would increase from 10.2 to 10.4%. We are going to correct the protocol in the third phase of KERCADRS to recheck FBS and HbA1c in the second blood sampling (recruitment). Thirdly, we could not distinguish type I from II diabetes as we did not review the individuals' medical records. Fourthly, although we tried to track all people who participated in phase 1, unfortunately, a significant number of them had changed their location between the two phases, and we were unable to contact them because mobile phones were not so popular in 2009–2011 or because some of them had died between the two phases. This may affect the incidence calculation in the study. However, an increase in the sample size to 10,000 in phase 2 strengthens the prevalence calculated in the study.

### Conclusion

There were promising signs of considerable reduction in the prevalence of pre-DM and undiagnosed DM and stability in the prevalence of DM in the 5-year interval between the two phases of the study. However, the finding that treatment is still ineffective in more than 55% of diagnosed DM individuals is a warning that the health care management system should take more effective measures in primary healthcare in urban areas. Early diagnosis and better management of diabetic cases are necessary to prevent further diabetes-caused morbidity and mortality in this area.

## Data Availability Statement

The original contributions presented in the study are included in the article/supplementary material, further inquiries can be directed to the corresponding author/s.

## Ethics Statement

The studies involving human participants were reviewed and approved by Ethics Committee of Kerman University of Medical Sciences (Ethical code: IR.KMU.REC.1392.405). Written informed consent to participate in this study was provided by the participants' legal guardian/next of kin.

## Author Contributions

HN, AM, and MSa designed the study. AM developed the analytic approach. HN, MSa, MF, and MSh participated in data collection. RA did the analysis with input from other authors. HN, MF, and MSh wrote the first draft of the paper. All authors contributed to finalizing of the paper.

## Funding

The KERCADR phase 2 study was a population-based cohort study implemented and funded by Kerman University of Medical Sciences (Grant No. KMU.93/310).

## Conflict of Interest

The authors declare that the research was conducted in the absence of any commercial or financial relationships that could be construed as a potential conflict of interest.

## Publisher's Note

All claims expressed in this article are solely those of the authors and do not necessarily represent those of their affiliated organizations, or those of the publisher, the editors and the reviewers. Any product that may be evaluated in this article, or claim that may be made by its manufacturer, is not guaranteed or endorsed by the publisher.
